# Alleviating psoriatic skin inflammation through augmentation of Treg cells via CTLA-4 signaling peptide

**DOI:** 10.3389/fimmu.2023.1233514

**Published:** 2023-09-25

**Authors:** Woo-Sung Lee, Kyung-Ho Nam, Jong Hoon Kim, Won-Ju Kim, Jeong Eun Kim, Eui-Cheol Shin, Gil-Ran Kim, Je-Min Choi

**Affiliations:** ^1^ Department of Life Science, College of Natural Sciences, Hanyang University, Seoul, Republic of Korea; ^2^ Department of Dermatology and Cutaneous Biology Research Institute, Gangnam Severance Hospital, Yonsei University College of Medicine, Seoul, Republic of Korea; ^3^ Department of Pediatrics, Stanford University School of Medicine, Stanford, CA, United States; ^4^ Department of Dermatology, Hanyang University College of Medicine, Seoul, Republic of Korea; ^5^ Hanyang Institute of Bioscience and Biotechnology, Hanyang University, Seoul, Republic of Korea; ^6^ Graduate School of Medical Science and Engineering, Korea Advanced Institute of Science and Technology (KAIST), Daejeon, Republic of Korea; ^7^ The Center for Viral Immunology, Korea Virus Research Institute, Institute for Basic Science (IBS), Daejeon, Republic of Korea; ^8^ Research Institute for Natural Sciences, Hanyang University, Seoul, Republic of Korea; ^9^ Research Institute for Convergence of Basic Sciences, Hanyang University, Seoul, Republic of Korea

**Keywords:** psoriasis, dNP2-ctCTLA-4, CTLA-4-Ig, Treg cells, IL-17A

## Abstract

Psoriasis is a chronic inflammatory skin disease characterized by hyperplasia of keratinocytes and immune cell infiltration. The IL-17-producing T cells play a key role in psoriasis pathogenesis, while regulatory T (Treg) cells are diminished during psoriatic inflammation. Current psoriasis treatments largely focus on IL-17 and IL-23, however, few studies have explored therapeutic drugs targeting an increase of Treg cells to control immune homeostasis. In this study, we investigated the effects of a cytotoxic T lymphocyte antigen-4 (CTLA-4) signaling peptide (dNP2-ctCTLA-4) in Th17, Tc17, γδ T cells, Treg cells *in vitro* and a mouse model of psoriasis. Treatment with dNP2-ctCTLA-4 peptide showed a significant reduction of psoriatic skin inflammation with increased Treg cell proportion and reduced IL-17 production by T cells, indicating a potential role in modulating psoriatic skin disease. We compared dNP2-ctCTLA-4 with CTLA-4-Ig and found that only dNP2-ctCTLA-4 ameliorated the psoriasis progression, with increased Treg cells and inhibited IL-17 production from γδ T cells. *In vitro* experiments using a T cell-antigen presenting cell co-culture system demonstrated the distinct mechanisms of dNP2-ctCTLA-4 compared to CTLA-4-Ig in the induction of Treg cells. These findings highlight the therapeutic potential of dNP2-ctCTLA-4 peptide in psoriasis by augmenting Treg/Teff ratio, offering a new approach to modulating the disease.

## Introduction

Psoriasis is a chronic inflammatory skin disease that affects approximately 2-3% of adults worldwide (1). Clinical features of psoriasis present as erythematous plaques with silvery-scale (2). It is characterized by systemic inflammation, which is associated with comorbid diseases such as arthritis, inflammatory bowel diseases, and cardiovascular diseases (3). The pathogenesis of psoriasis is complex and involves the infiltration of various immune cells, such as T cells and dendritic cells, into the skin (4). The activation of IL-17-secreting T cells through the IL-23-IL-17A axis plays a crucial role in the exacerbation of inflammation. Pro-inflammatory cytokines such as TNF-α, IL-6, and IL-1β secreted from keratinocytes, as well as effector cytokines such as IL-12 and IL-23 secreted from dendritic cells, contribute to the activation of Th17 and Tc17 cells (5, 6). In addition, innate lymphoid cell 3 (ILC3) and γδ T cells are also involved in skin inflammation by secreting IL-17A cytokine (7–12).

Psoriasis treatment strategies are determined based on the severity of the patient (13–15). Topical steroids and/or calcipotriol are commonly used to treat mild cases. For moderate-to-severe patients, systemic therapy including cyclosporin A, methotrexate, and narrow-band ultraviolet B is administered, and more recently, biologics and small molecule drugs have been used to target specific molecules involved in inflammation (16). These drugs include secukinumab and ixekizumab, which target the IL-17A cytokine, and guselkumab and risankizumab, which simultaneously inhibit IL-23 by targeting the p19 molecule (2, 3, 17–19).

Regulatory T (Treg) cells play a crucial role in maintaining immune homeostasis and regulating inflammatory responses (20, 21). These cells can be divided into two subsets: Natural Treg cells that develop in the thymus and induced Treg cells that are induced in the periphery and express the transcription factor Forkhead box P3 (Foxp3), which confers immunosuppressive function (20). Treg cells suppress effector T cell responses by depleting IL-2 signaling through high CD25 (IL-2Rα) expression, regulating co-stimulatory signaling through constitutive expression of CTLA-4, and secreting suppressive cytokines such as IL-10 (22–24). Defects in the number or suppressive function of Treg cells have been implicated in autoimmune diseases, including type 1 diabetes, multiple sclerosis, systemic lupus erythematosus, and psoriasis (25–29). The conversion of Treg cells to IL-17A-secreting cells that have lost their suppressive function has been shown in several autoimmune diseases including psoriasis (30–32). In animal models of psoriasis, depletion of Treg cells results in increased disease severity (33–35), highlighting their crucial role in disease regulation. Despite this knowledge, there are currently no treatments available specifically targeting Treg cell augmentation in psoriasis. In this study, we investigated a synthesized peptide that combines the signaling domain of CTLA-4 with a cell-penetrating peptide (CPP) to promote the generation of Foxp3^+^ Treg cells during psoriatic skin inflammation. Our findings indicate that the dNP2-ctCTLA-4 peptide holds promise as a therapeutic agent for psoriasis by inducing endogenous Treg cells *in vivo*.

## Results

### Induction of Foxp3^+^ Treg cells by dNP2-ctCTLA-4 peptide

**Figure 1:**
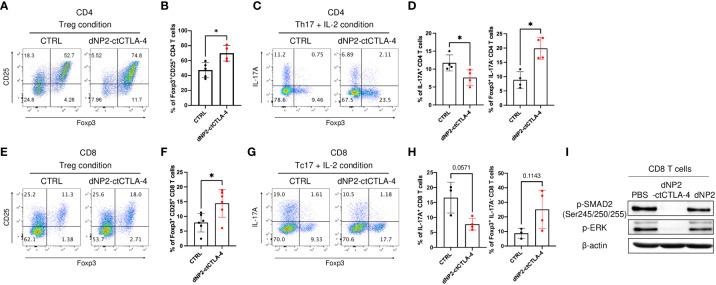
dNP2-ctCTLA-4 effectively induces Foxp3^+^ Treg cells in both CD4 and CD8 T cells. **(A-D)** Mouse naïve CD4 T cells were stimulated with anti-CD3/CD28 mAb in the presence or absence of dNP2-ctCTLA-4 for 3 or 4 days. Representative flow cytometric dot plot **(A)** and bar graphs **(B)** of expression of CD25 and Foxp3 under Treg differentiation condition (n=4~5). Representative flow cytometric dot plot **(C)** and bar graphs **(D)** of expression of IL-17A and Foxp3 under Th17 with IL-2 differentiation condition (n=4). **(E-H)** Mouse naïve CD8 T were stimulated with anti-CD3/CD28 mAb in the presence or absence of dNP2-ctCTLA-4 for 3 days. Representative flow cytometric dot plot **(E)** and bar graphs **(F)** of expression of CD25 and Foxp3 under Treg differentiation condition (n=6). Representative flow cytometric dot plot **(G)** and bar graphs **(H)** of expression of IL-17A and Foxp3 under Tc17 with IL-2 differentiation condition (n=3-4). **(I)** Mouse naïve CD8 T cells were stimulated with anti-CD3/CD28 mAb and TGF-β in the presence of dNP2-ctCTLA-4 or dNP2 peptide. The lysate was analyzed by immunoblotting of the p-SMAD2 linker region and p-ERK. Data are presented as mean ± S.D. Statistical significance was determined by the Mann-Whitney test. n.s. = nonsignificant, *p < 0.05.

Previously we generated the recombinant protein and a synthetic peptide of the cytoplasmic domain of the cytotoxic T lymphocyte antigen-4 (CTLA-4) in conjugation with a CPP dNP2 (dNP2-ctCTLA-4) (36–38). This protein and peptide showed successful immune modulatory effects on T cell activation during autoimmune or allergic disease with an increase of Foxp3 expressing Treg cells (36, 38–40). Building on this knowledge, we aimed to investigate whether the dNP2-ctCTLA-4 could be effective in treating psoriasis with increased Treg cells. As both Th17 (CD4) and Tc17 (CD8) cells are important pathogenic cells in psoriasis, we examined the functional effects of the dNP2-ctCTLA-4 peptide on mouse CD4 and CD8 T cells *in vitro*. In our experiments, we cultured TCRαβ^+^CD4^+^CD25^-^CD62L^high^CD44^low^ naïve CD4 T cells under Treg condition (TGF-β, IL-2) with or without the peptide. The results demonstrated a significant increase in CD25^+^Foxp3^+^ Treg cells when the peptide was present (**Figures 1A, B**). Furthermore, when we cultured these naïve CD4 T cells under Th17+IL-2 conditions (TGF-β, IL-6, IL-1β, IL-23, IL-2), dNP2-ctCTLA-4 exhibited inhibitory effects on IL-17 production while simultaneously increasing Foxp3 expression (**Figures 1C, D**). Similar observations were made with TCRαβ^+^CD8^+^CD25^-^CD62L^high^CD44^low^ naïve CD8 T cells, which were differentiated under Treg condition (TGF-β, IL-2) in the presence of dNP2-ctCTLA-4. The peptide treatment led to a significant increase in CD25^+^Foxp3^+^ CD8 T cells (**Figures 1E, F**). Moreover, when naïve CD8 T cells were cultured under Tc17+IL-2 conditions (TGF-β, IL-6, IL-1β, IL-23, IL-2), the peptide demonstrated a reduction in IL-17 production while promoting Foxp3 expression (**Figures 1G, H**). To investigate whether dNP2-ctCTLA-4 can intrinsically increase TGF-β mediated Foxp3 expression, naïve CD8 T cells were stimulated with anti-CD3 and TGF-β in the presence or absence of dNP2-ctCTLA-4 peptide. The dNP2-ctCTLA-4 peptide reduced SMAD2 linker region phosphorylation and the amount of p-ERK. This result is consistent with our previous research confirmed in the splenocytes (38), suggesting that dNP2-ctCTLA-4 peptide intrinsically enhances TGF-β signaling in CD8 T cells, leading to an increase in Foxp3^+^ T cells (**Figure 1I**). In summary, our findings demonstrate that the dNP2-ctCTLA-4 peptide has the ability to induce Foxp3 expression in both CD4 and CD8 T cells under various stimulatory conditions. These results suggest the therapeutic potential of the peptide in modulating Th17/Tc17 responses associated with psoriasis.

### Amelioration of psoriasis-like skin inflammation by dNP2-ctCTLA-4

**Figure 2:**
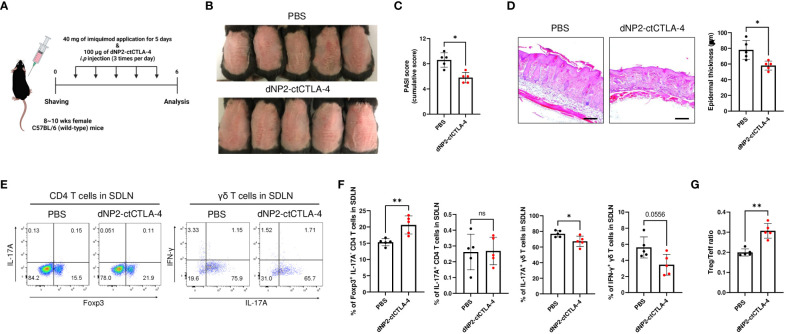
dNP2-ctCTLA-4 ameliorates psoriatic skin inflammation, accompanied by an increase in Foxp3^+^ CD4 T cells. **(A)** Experimental scheme of drug treatment in the imiquimod-induced psoriasis murine model. **(B)** Comparison of representative gross back skin phenotypes after the last IMQ application between the control and dNP2-ctCTLA-4 treated groups (n=5 per group). **(C)** Cumulative PASI scores (erythema, thickness, scaling). **(D)** Histological analysis of H&E-stained back skin samples (left, x100 magnification, scale bar = 200 μm) and epidermal thickness measured by ImageJ software (right). **(E)** Representative dot plots and **(F)** bar graphs of Foxp3, IL-17A, IFN-γ expression of isolated lymphocytes (CD4 T cells, γδ T cells) in SDLN. **(G)** The Treg/Teff ratio in SDLN. Data are presented as mean ± S.D. Statistical significance was determined by Mann–Whitney test. n.s., nonsignificant; *p < 0.05, **p < 0.01.

To assess the potential of the dNP2-ctCTLA-4 peptide in inducing Treg cells and alleviating psoriatic skin inflammation, we conducted *in vivo* experiments using the well-established imiquimod (IMQ)-induced psoriasis mouse model (41), which closely resembles human psoriasis (42). The IMQ-induced psoriasis mouse model was established by daily application of IMQ to the back skin of mice until day-5. Concurrently, the mice received intraperitoneal administration of 100 μg of the dNP2-ctCTLA-4 peptide three times daily from day-1 to 5 (**Figure 2A**). Our findings demonstrated that the dNP2-ctCTLA-4 peptide significantly inhibited the progression of psoriasis (**Figure 2B**) and effectively improved the psoriasis area and severity index (PASI) score (**Figure 2C**). Histological analysis of dorsal tissue samples revealed a reduction in epidermal hyperplasia and immune cell infiltration (**Figure 2D**). Flow cytometry analysis of skin-draining lymph nodes (SDLN) showed a notable increased in the proportion of Foxp3^+^ CD4 T cells, accompanied by a reduction in IL-17A-producing γδ T cells (**Figures 2E, F**). Taken together, these results highlight the potential of the dNP2-ctCTLA-4 peptide to induce Foxp3^+^ Treg cells *in vivo*, consequently ameliorating psoriasis-like skin inflammation. This induction of Treg cells leads to a significantly increased Treg/Teff ratio (**Figure 2G**), underscoring the therapeutic efficacy of the dNP2-ctCTLA-4 peptide in the treatment of psoriatic skin inflammation.

### Suppression of psoriatic skin inflammation by dNP2-ctCTLA-4, but not CTLA-4-Ig

**Figure 3:**
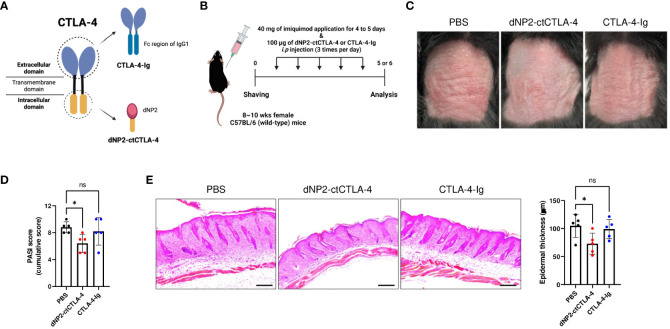
dNP2-ctCTLA-4 effectively improves psoriatic skin inflammation, while CTLA-4-Ig does not. **(A)** Schematic images of dNP2-ctCTLA-4 and CTLA-4-Ig utilizing each domain of CTLA-4. **(B)** Experimental scheme of drug treatment in the IMQ-induced psoriasis murine model. **(C)** Comparison of representative back skin phenotypes after last IMQ application between dNP2-ctCTLA4 and CTLA4-Ig treated group (n=5 per group). **(D)** Cumulative PASI scores (erythema, thickness, scaling). **(E)** Histological analysis of H&E-stained back skin samples (left, x100 magnification, scale bar = 200 μm) and epidermal thickness measured by ImageJ software (right). Data are presented as mean ± S.D. Statistical significance was determined by the Kruskal-Wallis test. n.s., nonsignificant; *p < 0.05.

CTLA-4-Ig (Abatacept) is a US Food and Drug Administration (FDA)-approved drug primarily used for rheumatoid arthritis treatment. It consists of the extracellular domain of CTLA-4 fused with the Fc region of an immunoglobulin (43, 44). By binding to B7 molecules, CTLA-4-Ig effectively inhibits the co-stimulatory signal of T cells, thereby preventing their activation (45). In contrast, the dNP2-ctCTLA-4 peptide utilized in our study employs the cytoplasmic domain of CTLA-4 to modulate intracellular signaling and result in Foxp3-expressing Treg cells. Considering the distinct domains of CTLA-4 targeted by these two agents (**Figure 3A**), we hypothesized that they would exhibit different mechanisms of action in treating psoriasis. To evaluate this hypothesis, equal doses of CTLA-4-Ig and dNP2-ctCTLA-4 peptide were administered to mice with IMQ-induced psoriatic skin inflammation, following the same protocol as in the previous experiment (**Figure 3B**). As shown in the figure, CTLA-4-Ig did not effectively inhibit psoriatic skin inflammation, while the dNP2-ctCTLA-4 peptide significantly reduced psoriasis symptoms and PASI score (**Figures 3C, D**). Additionally, only the group treated with the dNP2-ctCTLA-4 peptide demonstrated a decrease in epidermal layer thickness and infiltration of inflammatory cells (**Figure 3E**). These compelling findings suggest that the dNP2-ctCTLA-4 peptide, which specifically targets the intracellular signaling events related to the cytoplasmic domain of CTLA-4, would be more effective in regulating psoriatic skin inflammation compared to CTLA-4-Ig. The differential mechanisms of action of these two agents provide further insights into their distinct therapeutic potential for managing psoriasis.

### Induction of CD25^+^ Foxp3^+^ Treg cells and suppression of IL-17A-producing effector T cells by dNP2-ctCTLA-4, but not CTLA-4-Ig

**Figure 4:**
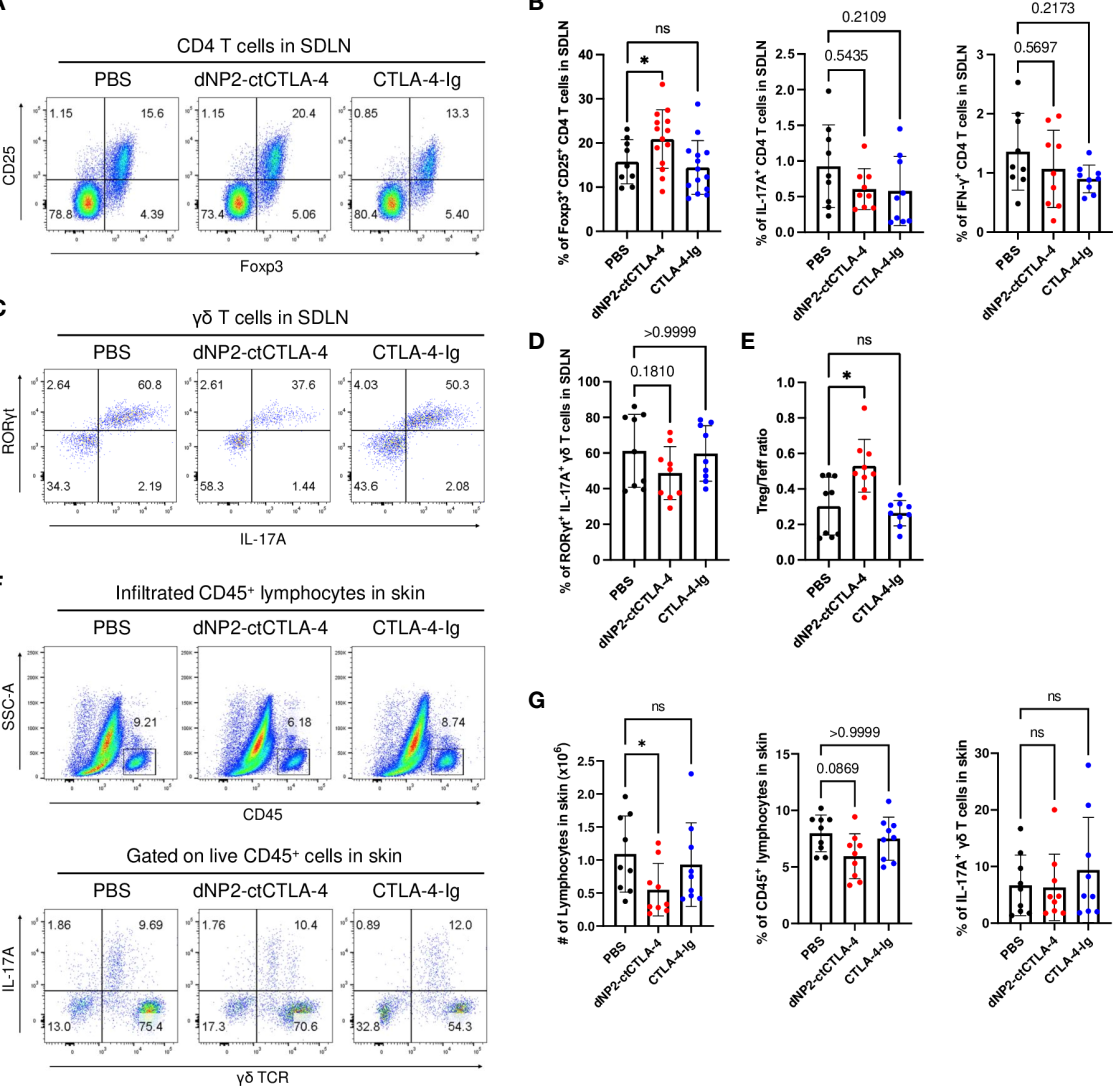
dNP2-ctCTLA-4 increases CD25^+^ Foxp3^+^ Treg cells in the SDLN of the mice, whereas CTLA-4-Ig does not. **(A)** Representative dot plots and **(B)** bar graphs of flow cytometric analysis in SDLN to identify CD25^+^ Foxp3^+^ CD4 T cells (n=9-14 per group), IL-17A^+^ CD4 T cells (n=9 per group) and IFN-γ^+^ CD4 T cells (n=9 per group). **(C)** Representative dot plots and **(D)** bar graphs of flow cytometric analysis in SDLN to identify IL-17A^+^ RORγt^+^ γδ T cells (n=9 per group). **(E)** The Treg-Teff ratio in SDLN (n=9 per group). **(F)** Representative dot plots and **(G)** bar graphs of flow cytometric analysis in the skin tissue to identify infiltrated lymphocytes, and IL-17A^+^ γδ T cells (n=9 per group). Data are presented as mean ± S.D. Statistical significance was determined by the Kruskal-Wallis test or Brown-Forsythe and Welch ANOVA tests. n.s., nonsignificant; *p < 0.05.

To investigate the impact of dNP2-ctCTLA-4 peptide and CTLA-4-Ig on lymphoid cells in psoriasis-like skin inflammation, we conducted a flow cytometry analysis. Intriguingly, the dNP2-ctCTLA-4 peptide significantly induced CD25^+^ Foxp3^+^ Treg cells, whereas CTLA-4-Ig could not in SDLN (**Figures 4A, B**). Neither treatment showed a statistically significant tendency to inhibit the production of IL-17A and IFN-γ in CD4 T cells (**Figure 4B**). Although the dNP2-ctCTLA-4 peptide resulted in a slightly reduced tendency in the proportion of IL-17A-producing γδ T cells (**Figures 4C, D**), it exhibited a significantly increased Treg/Teff ratio compared to CTLA-4-Ig, confirming its potential as an effective regulator of psoriatic skin inflammation (**Figure 4E**). Consistent with these findings, the dNP2-ctCTLA-4 peptide also demonstrated a significant reduction in the absolute cell number of infiltrated CD45^+^ lymphocytes into the skin tissue, as compared to control group (**Figures 4F, G**). However, it is worth noting that neither CTLA-4-Ig nor the dNP2-ctCTLA-4 peptide appeared to reduce the proportion of IL-17^+^ γδ T cells in the skin. Overall, this result suggests that CTLA-4-Ig might not be an effective molecule for controlling psoriatic skin inflammation, whereas the dNP2-ctCTLA-4 peptide could serve as a promising novel therapeutic option, as it exhibits a mechanism of increasing the Treg/Teff ratio.

### Distinct mechanisms of action by dNP2-ctCTLA-4 and CTLA-4-Ig during Th17 and Treg differentiation in T cell -antigen presenting cells (APC) coculture system

**Figure 5:**
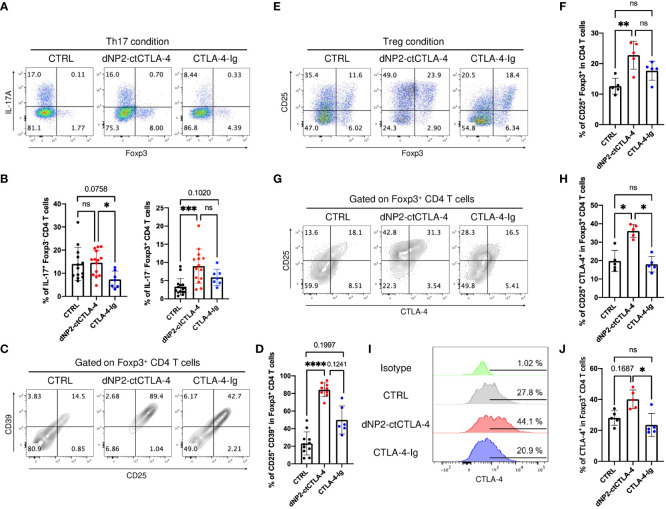
dNP2-ctCTLA-4 and CTLA-4-Ig exhibit distinct functional effects on T cell activation and differentiation. **(A-D)** Naïve CD4 T cells from 2D2 mice were stimulated with MOG_35-55_ peptide loaded by APCs in the presence of 3~5 μg/ml of dNP2-ctCTLA-4 or CTLA-4-Ig under Th17 condition. Representative flow cytometric dot plot **(A)** and bar graphs **(B)** of expression of IL-17 and Foxp3 under Th17 differentiation condition (n=6-14). Representative flow cytometric dot plot **(C)** and bar graphs **(D)** of Treg markers including CD25 and CD39 in Foxp3^+^ CD4 T cells (n=6-11). **(E-J)** Naïve CD4 T cells from 2D2 mice were stimulated with MOG_35-55_ peptide loaded by antigen-presenting cells in the presence of 3~5 μg/ml of dNP2-ctCTLA-4 or CTLA-4-Ig under Treg condition (n=5). Representative flow cytometric dot plot **(E)** and a bar graph **(F)** of Treg induction. Representative flow cytometric dot plot **(G)** and a bar graph **(H)** of Treg marker including CD25 and CTLA-4 in Foxp3^+^ CD4 T cells. Representative flow cytometric histogram **(I)** and bar graphs **(J)** of CTLA-4 expression in Foxp3^+^ CD4 T cells. Data are presented as mean ± S.D. Statistical significance was determined by the Kruskal-Wallis test. n.s., nonsignificant; *p < 0.05, **p<0.01, ***<0.001, ****<0.0001.

To gain further insights into the distinct mechanisms underlying T cell regulation by dNP2-ctCTLA-4 peptide and CTLA-4-Ig, we performed experiments utilizing FACS-sorted naïve CD4 T cells derived from 2D2 mice. These cells were stimulated with MOG_35-55_ peptide in the presence of APCs to induce Th17 and Treg cells, while being exposed to either the dNP2-ctCTLA-4 peptide or CTLA-4-Ig. Our findings revealed noteworthy disparities between the two agents. Specifically, the dNP2-ctCTLA-4 peptide demonstrated induction of Foxp3^+^ Treg cells under Th17 differentiation conditions (IL-6, TGF-β), which was not observed with CTLA-4-Ig (**Figures 5A, B**). Intriguingly, CTLA-4-Ig exhibited a tendency to inhibit IL-17^+^ CD4 T cells, a response not observed with the dNP2-ctCTLA-4 peptide. We further examined key functional molecules in Treg functions including CD25 and CD39. Notably, the dNP2-ctCTLA-4 peptide significantly upregulated both CD25 and CD39 expression in Foxp3^+^ Treg cells compared to CTLA-4-Ig (**Figures 5C, D**). Moreover, the dNP2-ctCTLA-4 peptide induced CD25^+^ Foxp3^+^ Treg cells under Treg conditions, while CTLA-4-Ig failed to elicit a similar response (**Figures 5E, F**). We observed an increase in the CD25^high^ CTLA-4^high^ population within Foxp3^+^ Treg cells under Treg conditions when treated with the dNP2-ctCTLA-4 peptide, but not with CTLA-4-Ig (**Figures 5G, H**). Notably, the expression level of CTLA-4 tended to be increased in Foxp3^+^ CD4 T cells by the dNP2-ctCTLA-4 peptide, whereas CTLA-4-Ig did not elicit a similar effect (**Figures 5I, J**). Collectively, these findings highlight that the dNP2-ctCTLA-4 peptide induces Foxp3^+^ Treg cells with enhanced Treg characteristics, while CTLA-4-Ig suppresses Th17 responses. Thus, our results suggest that the mechanisms underlying the actions of CTLA-4-Ig and the dNP2-ctCTLA-4 peptide are fundamentally different which provides a plausible explanation for the divergent outcomes observed in the psoriasis experiments.

## Discussion

**Figure 6:**
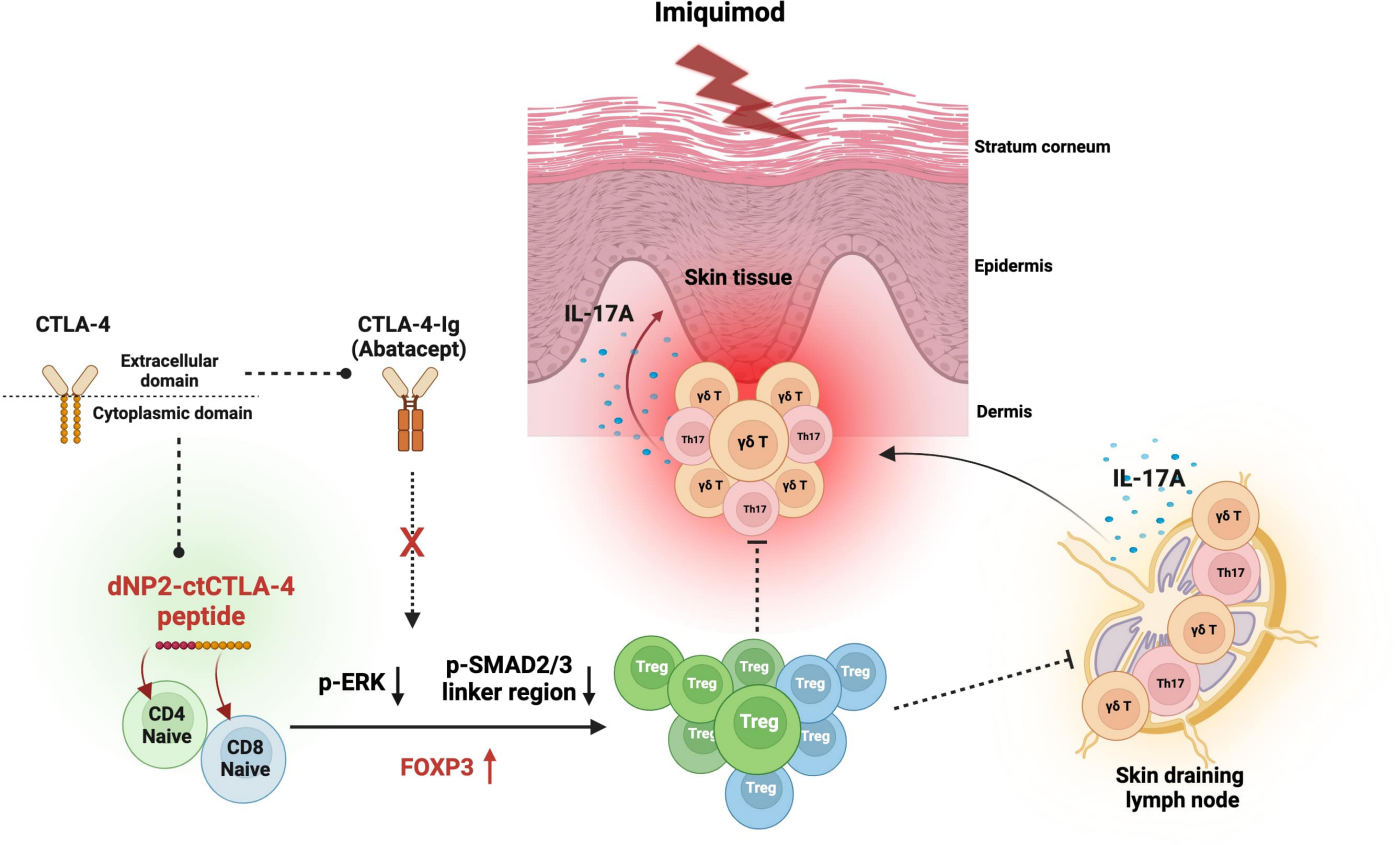
Graphical summary of the effects of dNP2-ctCTLA-4 on psoriatic skin inflammation. Diagram created with BioRender (BioRender.com). The dNP2-ctCTLA-4 peptide is composed of dNP2, a cell-penetrating peptide, and the cytoplasmic domain of CTLA-4. In contrast, CTLA-4-Ig (Abatacept) is a fusion protein that combines the Fc region of immunoglobulin IgG1 and the extracellular domain of CTLA-4. The dNP2-ctCTLA-4 peptide induces Foxp3 expression in both CD4 and CD8 T cells by inhibiting the p-ERK and p-SMAD2/3 linker regions. However, CTLA-4-Ig is incapable of inducing Foxp3^+^ Treg cells. The dNP2-ctCTLA-4 peptide can suppress IL-17A production in γδ T cells, Th17 and Tc17 cells while inducing Treg cells, thereby presumably alleviating psoriatic skin inflammation.

In this study, we conducted a comprehensive investigation into the therapeutic potential of a dNP2-ctCTLA-4 peptide utilizing an IMQ-induced psoriasis-like skin inflammation mouse model (**Figure 6**). Our findings substantiated the effectiveness of dNP2-ctCTLA-4, which employs a CPP fused with the cytoplasmic domain of CTLA-4. The peptide demonstrated its remarkable efficacy in inducing Foxp3-expressing T cells in both CD4 and CD8 T cells by inhibiting the p-ERK and the p-SMAD2/3 linker region *in vitro*. *In vivo* studies further demonstrated its ability to effectively ameliorate the progression of psoriatic skin inflammation and augment the population of Foxp3^+^ CD4 T cells. Notably, the Treg cells induced by the dNP2-ctCTLA-4 peptide exhibited heightened expression of functional markers, such as CD25, CD39, and CTLA-4 suggesting the induction of potent suppressive Treg cells. In contrast, CTLA-4-Ig, which employs the extracellular domain of CTLA-4, displayed a propensity to reduce Treg cell populations *in vitro* and provided ineffective in inhibiting psoriasis progression. Our findings shed light on Treg induction as a novel strategy for mitigating psoriasis-like skin inflammation and underscore the potential of dNP2-ctCTLA-4 as a promising therapeutic agent for autoimmune diseases, including psoriasis.

Psoriasis, a chronic inflammatory skin disease, is characterized by an immunological imbalance, resulting in the excessive secretion of IL-17A cytokine. This dysregulation occurs through the IL-23/IL-17A axis and involves multiple immune cell types. While IL-17-secreting αβ T-cells (Th17, Tc17) are the major contributor to pathogenesis in human psoriasis patients, γδ T cells are primarily responsible for inflammatory responses in mouse models (46–48). The γδ T cells expressing the Vγ4 and Vγ6 chain are found in the dermal layer of skin tissue (49–51) and known to secrete IL-17A in response to stimuli such as IL-1β and IL-23 (52, 53). They also migrate to inflamed areas by expressing C-C motif chemokine receptor 6 (CCR6) on their surface (54–56). Although there is evidence of Treg cells suppressing the activation of γδ T cells (57–59), the interplay between these cell types remains relatively unexplored. In this study, we demonstrated that dNP2-ctCTLA-4 peptide induces Treg cells in an inflammatory environment. It effectively suppresses IL-17A production by γδ T cells in SDLN, consequently reducing immune cell infiltration into the skin tissue. Furthermore, the dNP2-ctCTLA-4 peptide promotes the generation of Foxp3^+^ Treg cells while inhibiting IL-17A production during Th17 and Tc17 differentiation *in vitro*. In our previous report, we confirmed that the dNP2-ctCTLA-4 peptide can be delivered to T cells (36, 60) and inhibit T cell activation. We also demonstrated the peptide interferes with PKC-η to increase Treg cells, inhibiting progression of experimental autoimmune encephalomyelitis (EAE) (38). In the induced psoriasis model involving the ear, our findings once again demonstrated the efficacy of the dNP2-ctCTLA-4 peptide in effectively suppressing psoriatic skin inflammation (**Supplementary Figures S1A, B** in **Supplementary Material**). Notably, we observed a significant reduction in *Il17a* mRNA expression within the skin (**Supplementary Figure S1C** in **Supplementary Material**). Furthermore, we observed that the activation of γδ T cells, which are known to be activated by IL-1β and IL-23, could be effectively inhibited by the dNP2-ctCTLA-4 peptide (**Supplementary Figures S1D, E** in **Supplementary Material**). Therefore, we have expected that dNP2-ctCTLA-4 peptide could regulate both IL-17-secreting αβ T cells and IL-17A^+^ γδ T cells. These results provide compelling evidence that the dNP2-ctCTLA-4 peptide acts as a potent regulator of immune responses in the context of psoriatic skin inflammation. Its ability to attenuate the expression of IL-17A by Th17 and Tc17, a critical driver of psoriasis pathology, and inhibit the activation of γδ T cells further support its therapeutic potential in this disease.

CTLA-4 (CD152) is an immune checkpoint molecule that plays a crucial role in regulating T cell activity by competing with CD28 signaling to bind to the B7 molecules expressed on the surface of APCs (61–64). CTLA-4-Ig (Abatacept), a drug that combines the extracellular domain of CTLA-4 with the Fc region of an immunoglobulin, has been FDA-approved as a treatment for rheumatoid arthritis, active psoriatic arthritis. Our findings showed that only the dNP2-ctCTLA-4 peptide was effective in increasing Treg cells and Treg/Teff ratio, while CTLA-4-Ig did not show any therapeutic effect. In our T cell-APC coculture experiment, we observed that CTLA-4-Ig successfully suppressed antigen-specific T cell activation. However, when it came to alleviating psoriatic skin inflammation, CTLA-4-Ig did not yield the same results as the dNP2-ctCTLA-4 peptide. This discrepancy may be attributed to the fact that the IMQ-induced psoriasis model predominantly relies on the involvement of γδ T cells. Additionally, several studies have shown that CTLA-4-Ig reduces Treg cells (65, 66) and combination therapies involving rapamycin, which induces Treg cells, have been attempted to overcome this limitation (67). Clinically, CTLA-4-Ig has not been successful in treating psoriasis. Although CTLA-4-Ig was studied in a phase 1 clinical trial for patients with psoriasis vulgaris (NCT00306878, NCT0027722); the study was discontinued in 2011, and no results have been reported. While CTLA-4-Ig is used in patients with psoriatic arthritis, it only had a moderate impact on psoriasis symptoms (68). Moreover, it has been reported that CTLA-4-Ig failed to prevent psoriasis relapse after discontinuation of Ustekinumab treatment (69). Obviously, the cytoplasmic and extracellular domains of CTLA-4 play distinct roles in modulating inflammation, each operating through different mechanisms. The cytoplasmic domain of CTLA-4 encompasses various functional residues, including a crucial lysine residue that interacts with PKC-η protein (70). Our previous study demonstrated that the mutant form of the lysine residue of dNP2-ctCTLA-4 peptide failed to induce Foxp3^+^ Treg cells and ameliorate the progression of EAE disease, whereas the wild-type form of dNP2-ctCTLA-4 peptide effectively regulated EAE by inducing Treg cells (38). The dNP2-ctCTLA-4 peptide enhances TGF-β-Smad2/3 signaling, leading to increased Foxp3 expression in CD4 T cells through its binding with PKC-η protein. These findings highlight the importance of considering the differential mechanisms of action when designing therapies targeting CTLA-4 for the management of psoriasis.

Biologics have emerged as effective treatments for psoriasis, targeting specific cytokines, such as IL-17A and IL-23 (17, 71). However, despite their success, there are still some non-responders (72, 73) and currently available biologics do not offer a complete cure for psoriasis, often leading to relapse upon discontinuation. Treg cells play a critical role in maintaining immune tolerance and prevent autoimmune diseases. In a notable study, it was demonstrated that inducing Treg cells by topical application of short-chain fatty acids was shown to inhibit psoriatic skin inflammation reducing *Il17a* mRNA levels while increasing *Foxp3* and *Il10* mRNA levels in the skin (74). Moreover, the vitamin D analog, Maxacalcitol, significantly inhibits IMQ-induced psoriasiform dermatitis and reduces IL-17A mRNA expression in the skin, while also increasing Foxp3^+^ Treg cells (75). These findings support the notion that inducing Treg cells could serve as an effective treatment strategy for psoriasis, offering sustained therapeutic efficacy without recurrence after the cessation of therapy. Therefore, given the potential of Treg cells in regulating immune responses and the observed benefits of Treg cell induction in psoriasis, there is a promising opportunity to develop therapies that specifically target and modulate Treg cells, providing a novel approach for the treatment of psoriasis and other autoimmune conditions. Furthermore, the combination therapy of IL-17 or IL-23 blockade with Treg-inducing molecules such as dNP2-ctCTLA-4 peptide may hold therapeutic potential for more complete regulation of the disease.

Taken together, our findings suggest that the dNP2-ctCTLA-4 peptide holds promise as a novel therapeutic approach for psoriasis, offering the potential to modulate immune responses, suppress inflammation, and mitigate the pathogenic effects associated with controlling IL-17A producing Th17, Tc17, and γδ T cells via increasing Treg cells. Further research is warranted to fully elucidate the underlying mechanisms and explore the translational potential of the dNP2-ctCTLA-4 peptide as a targeted treatment for psoriasis.

## Materials and methods

### Mice

Female C57BL/6 mice aged 8 to 10 weeks were purchased from Daehan Biolink (DBL, Korea), while 2D2 TCR-transgenic mice were obtained from the Jackson Laboratory (Bar Harbor, ME, USA). The mice were housed and bred in a specific pathogen-free animal facility at Hanyang University under controlled conditions of constant temperature (21 ± 1°C), humidity (50 ± 5%), and a 12-hour light/dark cycle, and provided with regular chow and autoclaved water. All mouse experimental procedures were carried out in accordance with the guidelines of the Institutional Animal Care and Use Committees of Hanyang University (Animal Experiment Approval Number: 2022-0002A) and all mice studies were randomized in a blinded manner in psoriasis mouse models.

### Imiquimod-induced psoriasis-like mouse model

To induce psoriasis, 8- to 10-week-old female C57BL/6 mice purchased from Daehan Biolink (DBL, Korea) were used. On Day 0, hair was removed from the dorsal skin using a clipper and hair removal cream. Starting from Day 1, 40 mg of 5% Aldara Cream (3M Pharmaceuticals, Leicestershire, United Kingdom) was topically applied to the dorsal skin for 4 to 5 consecutive days. Psoriasis was induced under deep anesthesia with zoletil (30 mg/kg; Virvac, Carros, France) and rompun (10 mg/kg; Bayer Korea, Ansan, Korea). During disease induction, 100 μg of either dNP2-ctCTLA-4 or CTLA-4-Ig were administered intraperitoneally three times a day. On day 5 or 6, mice were sacrificed and the severity of inflammation in each tissue was assessed using flow cytometry (FACS) or histological analysis with hematoxylin and eosin (H&E) staining. Skin lesion severity was evaluated using the PASI scoring criteria, which considers three signs of erythema, scaling, and thickening, with a scale ranging from 0 (no symptoms) to 4 (very marked). To investigate the effect on ears, we applied 20 mg of 5% Aldara cream daily for six days, and administered 100 μg of protein via intraperitoneal injection. We measured the thickness of the ears using a micrometer (Mitutoyo, Kawasaki, Japan) every day and analyzed the data on day 7.

### Skin cell preparation

To acquire single cells, the fat was initially scraped off, and the dorsal skin tissue of the mouse was then separated into the epidermal and dermal layers at 37°C for 90 minutes using 2.4 U/ml of dispase II (04 942 078 001; Roche). After separation, the tissue was cut into small pieces and digested in DMEM media containing 1 mg/ml of collagenase D (11 088 866 001; Sigma-Aldrich), 0.2 mg/ml of DNase I (10 104 159 001; Sigma-Aldrich), and 2% FBS (S 001 01; Welgene) at 225 rpm for 1 hour at 37°C. The enzymatic reaction was stopped by adding 0.5 M ethylenediaminetetraacetic acid (EDTA), followed by filtering through a 40 µm cell strainer and washing with PBS. Single cells were finally obtained, and flow cytometry analysis was performed after antibody staining.

### Flow cytometry

Cells isolated from each tissue were analyzed using flow cytometry. Cells were restimulated with Cell Stimulation Cocktail (00-4975-03; eBioscience) to determine intracellular cytokine levels for 4 h at 37°C. To exclude dead cells, cells were stained with the Zombie Aqua Fixable Viability Kit (423101; BioLegend) at room temperature for 10 min. After washing with PBS, the surface proteins were stained with specific monoclonal antibodies for 20 min at 4°C. After washing with PBS, cells were fixed and permeabilized using Foxp3/Transcription Factor Staining Buffer Set (00-5523-00; eBioscience) for 30 min at room temperature. Finally, intracellular cytokine proteins were stained with monoclonal antibodies for 30 min at room temperature. FACS Canto II or FACS Symphony A3 was used to acquire data and FlowJo software version 10.8.0 was used to analyze the data.

### Histology

For histological analysis of mouse back skin samples, the tissues were fixed in 4% paraformaldehyde overnight at 4°C, followed by embedding in paraffin. Sections of the skin tissue blocks were prepared and stained with hematoxylin and eosin to reveal the cellular architecture. The resulting H&E-stained sections were then mounted with a cover glass using a mounting solution. Subsequently, the sections were observed under an inverted microscope (Leica DMi8, Leica Microsystems), and images were captured and analyzed using Image J software version 2.0.0

### *In vitro* T cell differentiation

Naïve CD4 T cells (TCRαβ^+^CD4^+^CD25^-^CD62L^high^CD44^low^) or naïve CD8 T cells (TCRαβ^+^CD8^+^CD25^-^CD62L^high^CD44^low^) were isolated from splenocytes of 8- to 10-week-old C57BL/6 mice using a FACSAria Fusion (BD Biosciences). Purified naïve CD4 T cells were activated with plate-bound anti-CD3 (2 μg/ml; 553057; BD Pharmingen) and anti-CD28 (2 μg/ml; 553294; BD Pharmingen) antibodies and differentiated with the following cytokine cocktails in a 96-well plate for 3 or 4 days: 20 U/ml of IL-2 (212-12; Peprotech), 2 ng/ml of TGF-β (240-B-002; R&D Systems) for Treg differentiation; or 5 μg/ml of anti-IL-4 (11B11; 16-7041-85; eBioscience), 5 μg/ml of anti-IFN-γ (XMG1.2; 16-7311-85; eBioscience), 30 ng/ml of IL-6 (406-ML; R&D Systems), 2 ng/ml of TGF-β, 20 ng/ml of IL-1β (401-ML-010; R&D Systems), 20 ng/ml of IL-23 (1887-ML-101; R&D Systems), 20 U/ml of IL-2 for Th17 differentiation. Additionally, cells were co-treated with 2 μM of dNP2-ctCTLA-4. Purified naïve CD8 T cells were activated with plate-bound anti-CD3 (3 μg/ml) and anti-CD28 (3 μg/ml) antibodies and differentiated with the following cytokine cocktails in a 96-well plate for 3 days: 25 U/ml of IL-2, 0.5 ng/ml of TGF-β for Treg differentiation; or 5 μg/ml of anti-IL-4, 5 μg/ml of anti-IFN-γ, 30 ng/ml of IL-6, 2 ng/ml of TGF-β, 20 ng/ml of IL-1β, 20 ng/ml of IL-23, 20 U/ml of IL-2 for Tc17 differentiation. Additionally, cells were co-treated with 2 μM of dNP2-ctCTLA-4.

### *In vitro* co-culture assay

Naïve CD4 T cells (CD4^+^TCR-Vβ11^+^CD25^-^CD62L^high^CD44^low^) were isolated from splenocytes of 8- to 10-week-old 2D2 mice using the CD4 T Cell Isolation Kit (Miltenyi Biotec) according to the manufacturer’s instructions, followed by FACS sorting. The antigen-presenting cells were irradiated with a 3500 rad gamma ray and seeded with the sorted naïve CD4 T cells at a 5:1 ratio. T cells were activated using 40 μg/ml of MOG_35-55_ (AnyGen, Korea), and differentiated using the following cytokine cocktails in a 96-well flat bottom plate for 3 days: 5 μg/ml of anti-IL-4 (11B11; 16-7041-85; eBioscience), 5 μg/ml of anti-IFN-γ (XMG1.2; 16-7311-85; eBioscience), 30 ng/ml of IL-6 (406-ML; R&D Systems), 2 ng/ml of TGF-β (240-B-002; R&D Systems) for Th17 differentiation; or 5 ng/ml of TGF-β for Treg differentiation. Additionally, cells were co-treated with dNP2-ctCTLA-4 or CTLA-4-Ig.

### Immunoblotting

Naïve CD8 T cells were isolated and stimulated with 5μg/ml of anti-CD3/CD28 mAb and 5 ng/ml of TGF-β at 37°C, 30 minutes. Afterward, the cells were washed with PBS and lysed with RIPA buffer (Cell Signaling, Beverly, MA) containing 1 μM of NaF and 1 μM of PMSF for 30 minutes on ice. Immunoblotting was performed on PVDF membranes (Bio-Rad) using the following primary antibodies: phospho-Smad2, phospho-ERK, and β-actin mouse mAb.

### Recombinant protein purification and peptide synthesis

dNP2-conjugated recombinant proteins were purified as previously described (36). Briefly, dNP2-ctCTLA-4 was purified by Ni-NTA affinity chromatography (Qiagen, Chatsworth, CA, USA) and desalted using a PD-10 Sephadex G-25 column (GE Healthcare, Uppsala, Sweden). To obtain highly purified proteins, an additional ion-exchange protein purification step was performed using SP Sepharose High Performance (GE Healthcare, Uppsala, Sweden). For the peptide form of dNP2-ctCTLA-4 (from AnyGen, Korea), the peptide was synthesized by solid-phase peptide synthesis as previously described (38). The purity of the final peptide products was more than 95%.

### RNA extraction and quantitative PCR

To extract RNA from ear tissue, we used TRI Reagent RT (Molecular Research Center, Inc.) following the manufacturer’s protocol. The extracted RNA was then quantified using a UV spectrophotometer (CellTAGen, Inc., Seoul, Korea). Subsequently, cDNA synthesis was performed using a ReverTra Ace qPCR RT Master Mix (Toyobo, Osaka, Japan). For quantitative RT-PCR analysis, we used iQ SYBR Green Supermix (Bio-Rad, Hercules, CA, USA) with a Bio-Rad CFX Connect real-time PCR detection system. To ensure accuracy, we normalized expression levels to CD45.

### Antibodies

The following monoclonal antibodies were used for cell surface staining: anti-mouse CD45 (30-F11; BioLegend), anti-mouse TCRβ (H57-597; eBioscience), anti-mouse TCRγδ (eBioGL3 (GL-3, GL3); eBioscience), anti-mouse Vγ1 (2.11; BioLegend), anti-mouse CD4 (RM4-5; BioLegend), anti-mouse CD25 (PC61; BioLegend), anti-mouse CD27 (LG.3A10; BD Biosciences), anti-mouse CD39 (Duha59; BioLegend), anti-mouse CD44 (IM7; BioLegend), anti-mouse CD62L (MEL-14; eBioscience). The following antibodies were used for intracellular staining: anti-mouse Foxp3 (FJK-16s; eBioscience), anti-RORγt (Q31-415 378; BD Biosciences), anti-mouse IL-17A (eBio17B7; eBioscience), anti-mouse IFN-γ (XMG1.2; eBioscience), anti-mouse CTLA-4 (UC10-4B9; eBioscience).

### Statistical analysis

Statistical analysis was conducted using GraphPad Prism version 7.0 (GraphPad Software, San Diego, CA, USA). Mann–Whitney test, Kruskal–Wallis test and Brown-Forsythe and Welch ANOVA tests were employed for data analysis. The data are presented as mean ± S.D. or ± S.E.M., and p ≤ 0.05 was considered statistically significant. Additional information regarding sample size and specific statistical analyses can be found in the corresponding figure legend.

## Data availability statement

The original contributions presented in the study are included in the article/**Supplementary Material**. Further inquiries can be directed to the corresponding authors.

## Ethics statement

The animal study was approved by Institutional Animal Care and Use Committees of Hanyang University. The study was conducted in accordance with the local legislation and institutional requirements.

## Author contributions

J-MC designed and supervised the project. W-SL conducted and analyzed an experiment using a psoriasis mouse back skin model. K-HN designed comparative analysis study using dNP2-ctCTLA-4 and CTLA-4-Ig by an *in vitro* T cell-APC co-culture assay and also performed the Tc17 experiment. JHK and W-JK initiated this study by analyzing psoriasis mouse ear model experiment and *in vitro* γδ T cell experiments. G-RK provided support recombinant proteins and synthetic peptides and performed *in vitro* Th17, Treg differentiation, and immunoblotting experiment. JEK and E-CS provided valuable technical advice on the psoriasis mouse model and contributed insightful comments on the results. W-SL, K-HN, and G-RK prepared the original draft and J-MC revised the draft. All authors contributed to the article and approved the submitted version.
