# Supplementary material for: Alleviating psoriatic skin inflammation through augmentation of Treg cells via CTLA-4 signaling peptide
Source: Front Immunol. 2023 Sep 25;14:1233514. doi: 10.3389/fimmu.2023.1233514 (PMC10560854; doi:10.3389/fimmu.2023.1233514)
Supplement: Supplementary file 1 [file DataSheet_1.pdf]

Supplementary Material

Alleviating psoriatic skin inflammation through augmentation of Treg cells via CTLA-4 signaling peptide

Woo-Sung Lee[1,†], Kyung-Ho Nam[1,†], Jong-Hoon Kim[2,†], Won-Ju Kim[3,†], Jeong Eun Kim[4,5], Eui-Cheol Shin[6,7], Gil-Ran Kim[1,8,*], and Je-Min Choi[1,5,8,9,*]

*** Correspondence:**
Gil-Ran Kim, godqhr3079@hanyang.ac.kr.
Je-Min Choi, jeminchoi@hanyang.ac.kr.

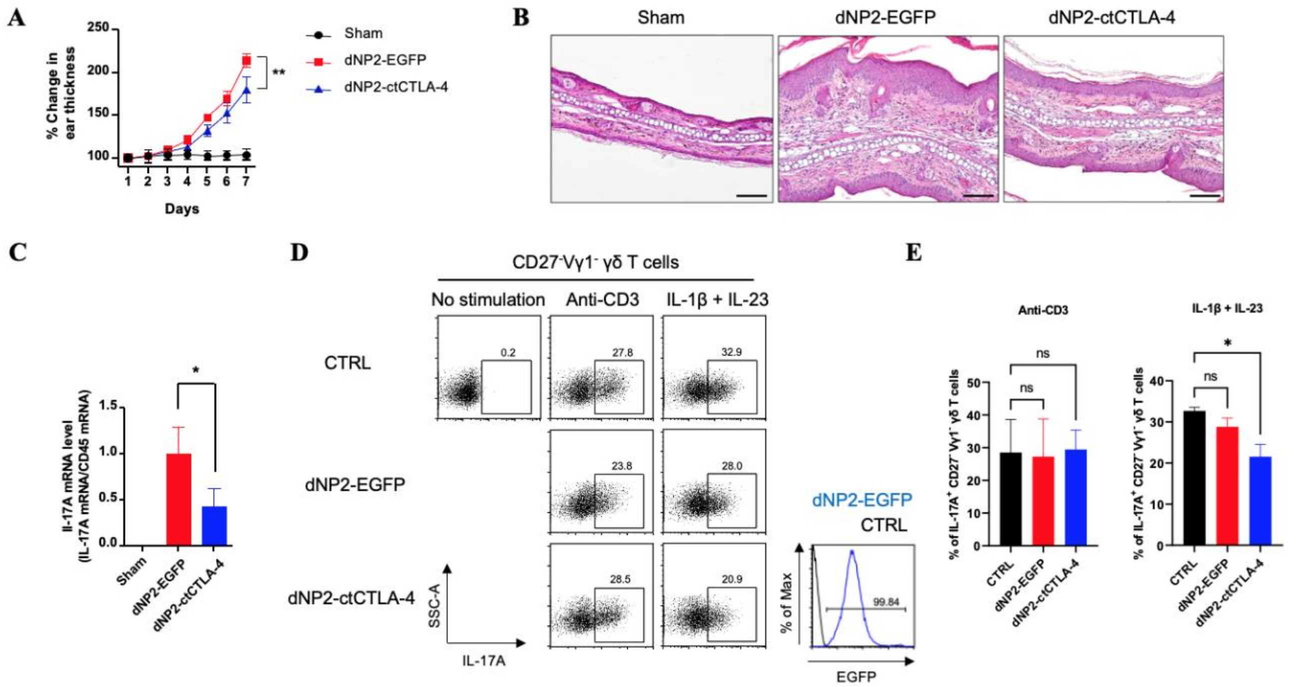

Figure S1. dNP2-ctCTLA-4 alleviates psoriasis-like skin inflammation and inhibits IL-17 production by γδ T cells. (A) Ear thickness measurement and thickness change of psoriasis-induced mice from day 1 to 7 (n=5). (B) Histological analysis of ear skin tissues (x100 magnification, scale bar = 200 μm). (C) IL-17A mRNA expression level in ear lesions. (D) Representative figure of inhibition experiment by dNP2-ctCTLA-4 under γδ T cell stimulation conditions *in vitro*. (E) Bar graphs of IL-17A^{+} cells in CD27^{-}Vγ1^{-} γδ T cells under TCR stimulation by anti-CD3 (left) and cytokine stimulation by IL-1β and IL-23 (right) (n=5). Data are presented as mean ± S.D. Statistical significance was determined by Mann–Whitney test. n.s. = nonsignificant, * $p < 0.05$, ** $p < 0.01$.
